# On the Physiological Action of Bromide of Potassium

**Published:** 1871-11

**Authors:** 


### On the Physiological action of Bromide of Potassium.

In the *Archives fur Heilkunde*, Heft 2, 1871, is an elaborate paper upon this subject by *G. I. Schouten*, an extended abstract of which is contained in *Allgemeine Medicinishe Central-Zeitung*, June and July, 1871. From this abstract we give a condensed translation, omitting the historical portion.

Grandeau's observation, that even small quantities of potash salts injected directly into the blood cause almost immediate death, has been confirmed by almost every observer, for following such injection comes heart-paralysis, with consequent cessation of the circulation and death, artificial respiration being powerless to re-excite the heart's action. On opening the thorax, if but a small amount of the potash salt has been used, spontaneous contractions are often visible, sometimes of the whole heart, more often of the auricle alone; if the dose has been a little larger, the ventricle is always found still, but excitable by electrical stimulation. When a very large amount of the salt has been injected, the whole heart is perfectly quiet and insusceptible to electrical excitation. If the thorax of a kitten be so opened that the heart's movements can be watched and a two per cent solution of bromide of potash be slowly injected into the jugular vein, the motion will be seen to become slower, the pauses longer, and at last the heart stands still, or at least merely fibrillar contractions take place, no full systole. During the injection, however slowly it may be made, slowing of the heart's movements and lengthening of the pauses can always be observed, never acceleration of the heart-beat, although immediately afterwards the frequency of the pulse plainly increases.

The quantity that can be injected into the jugular vein of a cat without causing death is very small, and depends upon the strength of the solution and rapidity of injection. Of a one per cent. solution, some 50 c. c. (0.5 grammes of the salt) were injected into the jugular of a cat without causing death, but then an hour was required for the injection; a few c. c. of two per cent. solution always was fatal. In the crural veins, however, 25 c.c. of the latter solution were injected without lethal effects; but when the repetition of the injection was attempted three hours afterwards, the animal died suddenly so soon as 6 c.c. had passed into the vein. Twenty-five c.c. of a three and a half per cent. solution were injected into the carotid and femoral arteries without killing the cat.

The injection of the chloride of potassium was attended with similar results, whilst the bromide of sodium was without influence.

In the case of dogs the effects were similar, but larger doses of the potash salts were required to kill. Thus, the injection of 3 c.c. of a six per cent. solution of bromide of potassium in the jugular vein of a dog caused immediate death, whilst 30 c.c. of a six per cent. solution of chloride of sodium were rapidly thrown into the same vein without disturbing the heart's action.

One cat bore without injury the subcutaneous injection of six grammes K. Br., whilst in another death followed two days after the similar use of the same quantity. Ten grammes produced death in one and a half hours and the chloride of potassium appeared even more powerful than the bromide. Six grammes of bromide of sodium did not appear to exert any influence whatever. When given by the stomach, six grammes of the bromide of potassium do not cause death in the cat, but 10 grammes are speedily fatal. The dog will bear the latter quantity well.

By small doses, voluntary motion and reflex activity were not perceptibly altered. With larger doses there were very evident weakness and uncertainty of movement, and animal sitting still, and only moving when strongly urged to it. Tremblings of the muscles only happened after injection of the salt into the blood, especially into the carotid artery. The observation that the cautious injection into the jugular produces immediately lengthening of the intervals without any increase of the rapidity ot the heart's motion, Dr. Schouten thinks it of great importance, as showing that the drug, when acting directly upon the heart, does not first produce excitement, and only after this paresis. The fact that much larger quantities are borne injected into the carotid than into the jugular, shows that the central nervous system is not so readily affected as the muscles. The experiments with the chloride of potassium and bromide of sodium show that Guttmann and Eulenberg were correct in attributing the action of bromide of potassium to the potash contained in it.

After all non-fatal doses of the bromide or chloride of potassium, even if so small as to have no perceptible influence on sensation or motion, there is constantly acceleration of the pulse. After injection into the blood or under the skin this increase came on quickly; on exhibition by the stomach, only after some time. The influence on the pulse is more apparent when the latter is slow than when it is rapid. When subcutaneous injection is used, the increase is almost in inverse ratio to the dose, being very much more marked after two to four grammes than after five to six. This does not occur, however, when the drug is exhibited by the mouth or thrown into the blood. If the animal survives, the increase of the pulse frequency passes off gradually, and is not followed by a falling of it below the normal rate. Bromide of sodium has no influence upon the frequency of the heart's action. Very large doses injected directly into the blood may indeed be followed by such increase, but it is so slight and fugitive as to be scarcely worthy of note.

3. *On the Influence on the Arterial Pressure.*—Kemmerich states that small doses of the potash salts increased not only the rapidity, but also the energy of the heart's action. He found the pulse fuller and stronger, and concluded that the potash salts, like other heart-poisons, had in small doses an exciting, in larger a paralyzing effect upon the heart, and that this paralysis was preceded by a stage of very strong stimulation of the heart-ganglia. Kemmerich did not, however, *prove* that the energy of the heart was increased. Even if the pulse felt more rapid and fuller, it is possible that the heart was really doing less than its normal work. Mere frequency of action is of no importance, and Dr. Schouten shows by experiment that just as *the pulse frequency rises so does the blood pressure fall.*

This is of great interest in connection with the therapeutical use of the drug. It was very difficult to reconcile Kemmerich's assertion that there was real excitation of the heart, with the clinically well-known quieting actions of the bromides.

In explaining the consentaneous increase in the rapidity of the heart's action and sinking of the blood pressure, the inter-dependence between the heart and blood-vessels, recently proven, is of great service. The nerves of the heart and of the blood-vessels exercise influence both upon the pulse frequency and arterial pressure. Is the blood-path anywhere enlarged, the arteries exert less resistance to the heart, and the blood pressure sinks, and at the same time, by the lessened blood pressure, the vagus centre is less stimulated, and the rapidity of the heart's action is increased.

The relation between the innervation of the heart and arteries is very complicated, and it is therefore very difficult to explain with certitude the causes of the observed weakening of pressure and increase of the pulse frequence. In order, however, to determine this, if possible, Dr. Schouten made various experiments. He found that after section of the vagi there was still increase in the rapidity of the heart's action. Immediately after the injection there was some slowing, but very soon the strokes began to grow more rapid. The pressure, on the contrary, was at first increased, and an hour after the injection had not fallen below the normal point. There is, however, a difficulty in this operation which cannot be surmounted. Division of both vagi and tracheotomy are very serious operations, which produce great disturbance in the system. One cannot, therefore, wait long after the operation. The fact that after the injection of the bromide of potassium, the pneumogastrics having been previously cut, the increased frequency quickly returns to the normal rapidity, gives ground for believing that if it had been possible to wait longer after the operation the increased frequency might not have occurred. The action of the bromide on the heart is then certainly not dependent upon the vagi nerves, although Dr. Schouten is by no means disposed to deny them all influence in the matter. The question, what influence the bromide exerts upon the innervation of the vessels and upon their tone, is not certainly answered; but Dr. S. believes that whilst positive proof is not forthcoming, it is very probable that relaxation occurs. When it is remembered that by the potash salts the voluntary muscles are injured in their functional power, the heart by even small amounts brought to a stand-still and its excitability destroyed, it seems most probable that the tone of the blood-vessels is lessened, even if the vaso-motor nerve-centres suffer no disturbance.

The action of the bromide, according to our author, happens in this way.

Under the influence of the bromide of potash a disturbance of nutrition occurs, so that in muscle and nerve the activity of tissue-changes is lessened. The heart activity naturally suffers from this, but so does also the innervation of the vagus and vaso-motor nerves, by the disturbance of which the rapidity of the heart-beats is increased, but not sufficiently to bring up the otherwise lessened arterial pressure. The calls on the heart are also lessened, owing to the lessened functional and chemical activities of the nerves.

By large doses the capability of action is so diminished, that in spite of the action on the heart's innervation the pulse frequency sinks. In cats, under these circumstances, death rapidly ensues. In this animal the tissue changes in health are so slight that they cannot be materially depressed without compromising life. In other animals in whom tissue-changes are more active, life can be maintained much longer. The main point here, however, is *that the increase of frequency of beat does not really mean increase in the work of the heart.*

That the bromide of potassium does lessen the activity of the tissue-changes was shown by the experiments of Kemmerich After twenty days, a hound fed on cooked flesh with a certian amount of potash salt weighed 1,275 grammes more than another which had received the same daily allowance, except that a soda salt was substituted for the potash salt, and a reversal in the use of the salts caused a reversal in the proportional weight of the dogs, dog No. 2 soon outstripping No. 1 when the former received the potash, the latter the soda.

Another proof lies in the diminished respiration, which Dr. Schouten has noticed as a constant result of the use of the bromide ofpotassium.

So also points the quieting influence which the bromide has upon abnormally energetic action of the heart and of the nerve-centres.

Whether the bromide acts in any way differently fromother salts of potash, Dr. Schouten says he cannot say. It is certain that all of the potash salts have a similar influence on the pulse frequency and blood pressure; but whether one salt acts more intensely than another is not yet decided. Dr. Sasse has already called attention to one thing which must have influence on this point, namely, the more or less quick separation of the salt by the kidneys. According to Rabuteau, the iodide and bromide of potassium differ very much in this particular, the bromide of potassium being separated much more slowly, so that after four weeks it could still be found in the urine. Surprised at this statement, Dr. S. made one or two experiments, finding the bromide in the ashes of the urine, in one case, five daysafter the injection of 0.5 grammes into the jugular vein of a cat, and absent on the sixth day; in another failing to find it four days after the injection of 28 grammes! (So printed; evidently a mistake.)—*New Remedies.*

